# Numerical Investigation on Multiple Resonant Modes of Double-Layer Plasmonic Grooves for Sensing Application

**DOI:** 10.3390/nano10020308

**Published:** 2020-02-11

**Authors:** Shuwen Chu, Qiao Wang, Li Yu, Huixuan Gao, Yuzhang Liang, Wei Peng

**Affiliations:** 1 School of Optoelectronic Engineering and Instrumentation Science, Dalian University of Technology, Dalian 116024, China; chuswdlut@163.com (S.C.); 1050027560@mail.dlut.edu.cn (L.Y.); shark@mail.dlut.edu.cn (H.G.); 2 School of Physics, Dalian University of Technology, Dalian 116024, China; yzliang@nju.edu.cn

**Keywords:** double-layer plasmonic grooves, multi-resonance sensing, surface plasmon polaritons

## Abstract

A high-performance multi-resonance plasmonic sensor with double-layer metallic grooves is theoretically constructed by introducing a polymethyl methacrylate groove with a numerical simulation method. Multiple resonance wavelengths can be generated at the oblique incidence, and the number and feature of resonant mode for sensing detection is different for various incident angles. Specifically, at the incident angle of 30°, the reflection spectrum exhibits two resonant dips, in which the dip at the wavelength of 1066 nm has an extremely narrow line width of ~4.5 nm and high figure of merit of ~111.11. As the incident angle increases, the electric dipole mode gradually weakens, but the surface plasmon resonance and cavity resonance mode are enhanced. Therefore, for an incident angle of 65°, three resonance dips for sensing can be generated in the reflection spectrum to realize three-channel sensing measurement. These double-layer plasmonic grooves have potential in the development of advanced biochemical surface plasmon polariton measurements.

## 1. Introduction

Since the discovery of extraordinary optical transmission through subwavelength aperture arrays on opaque metal films [1], the design of metal nanostructures and analysis of involved physical mechanisms have attracted enormous attention, and these studies have tremendously contributed to the development of surface plasmonic nanophotonics [2,3,4,5,6]. In the past few years, many applications on the basis of surface plasmon nanostructures have been proposed, such as sensors, superlens imaging [7,8,9], negative refractive effects [10,11,12], color filters [13,14,15,16,17], perfect absorbers [18,19,20] and the like. Surface plasmon sensors have developed into an advanced detection method because of their high sensitivity, wide detection range and easy miniaturization. The one dimensional groove array, a simple structure, has attracted attention as a micro-nano sensor, which is easy to integrate. However, structural parameters exert a significant impact on the performance of the device [21,22,23,24]. Compared to a single-layer groove structure, double-layer plasmonic grooves can more freely adjust the wavelength position. Meanwhile, it is more conducive to the coupling between various modes, generating new optical characteristics. Currently, most research involving groove sensors has concentrated on the near-infrared region. However, very few studies have been achieved on the visible region [25,26,27,28]. In addition, the resonant mode of a single-layer groove sensor possesses a relatively wide full wave at half maximum (FWHM), resulting in a low figure of merit (FOM) in the sensing detection and limiting its sensing application to some extent [29,30,31,32,33,34,35,36]. For example, Fu et al. theoretically explored nanohole arrays arranged in a hexagonal lattice with a sensitivity of 348 nm/RIU and a FOM of 34.8 [33]. Liu et al. proposed a network-type metasurface with multiple reflection bands, which achieved FWHM of 3 nm in the visible and near-infrared regions, and the FOM reached up to 68.57 [34]. Sharm et al. presented a self-referenced SPP sensor incorporating titanium oxide grating on thin gold film atop dielectric substrates in the optical communication band, and an average spectral sensitivity and Surface plasmon polaritons (SPP) curve width of 693.88 nm/RIU and 26.03 nm were achieved for the optimized values of grating variables [35]. Li et al. numerically and experimentally demonstrated a similar structure based on double-layered metal grating, but they tested only the reflection from the top surface with an FOM of 38 under normal incidence [36].

In order to overcome the shortcomings of a single-layer plasmonic groove structure in the sensing application, this paper proposes a novel structure in which multiple resonant modes of double-layer plasmonic grooves are induced by employing a prism. A double-layer groove structure is established with a polymethyl methacrylate groove array. The simulation results indicate that the designed structure possesses multiple resonances in the visible and near-infrared regions under an oblique incident angle. In the case of the incident angle 65°, wavelength positions of two resonant modes redshift with the increase of the refractive index of the surrounding environment. However, the wavelength position of another resonant mode remains constant, and its intensity has a change. Notably, for the incident angle 30°, the double-layer groove structure generates a sharp sub-radiant resonance with a narrow line width of 4.5 nm and a high FOM of up to 111.11. Additionally, there is a good linear approximation between wavelength position and ambient refractive index. Finally, the physical mechanisms of these resonances are further revealed by changing relevant structural parameters and analyzing corresponding electromagnetic field distribution.

## 2. Structures and Methods

The SPP is an electromagnetic surface wave that has the largest field strength at the surface and an exponential decay field perpendicular to the interface direction simultaneously. Exciting SPPs on the surface of metal and dielectric satisfies the following formula [37]:

(1) $$\frac{2\pi }{\lambda_{0}}\sin\theta +m\frac{2\pi }{P}=-\frac{2\pi }{\lambda_{0}}\sqrt{\frac{\varepsilon_{1}\varepsilon_{2}}{\varepsilon_{1}+\varepsilon_{2}}}=K_{\mathrm{SPPs}}$$

where *λ*_0_ and *θ* are wavelength and angle of incident light, respectively, *P* is the period of structure, *m* is diffraction order, *ε*_1_ and *ε*_2_ are relative permittivity of the dielectric material and *K*_spps_ represents the wave vector of the SPP wave. When the incident light meets the resonance condition, it converts energy into SPPs that propagate along the metal–dielectric interface.

**Figure 1:**
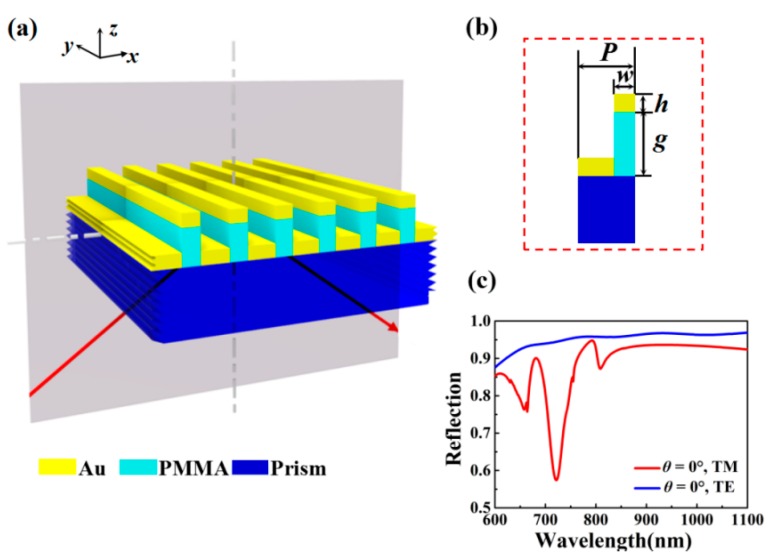
Schematics of the double-layer metallic groove structure. (**a**) Three-dimensional diagram of the designed structure consisting of two sets of metallic grooves upon the Kretschmann prism. (**b**) Grating diagram of one unit and corresponding structural parameters. (**c**) Typical optical spectra of the grooves for transverse magnetic (TM) and transverse electric (TE) polarized light under normal incidence.

Figure 1a,b illustrate the schematic of the proposed double-layer metallic groove structure. Two sets of metallic grooves were perpendicularly separated by high refractive index polymethyl methacrylate (PMMA) groove. There were different nanowell widths and the same nanowell thickness between these two set of grooves. The design of this structure is a reform of the classical Kretschmann model [38,39]. The resonant modes of the double-layer groove were excited by using a prism. The reflection spectrum of the designed nanostructure under normal incidence is shown in Figure 1c. It can be seen that there was no resonance response for transverse electric (TE) polarized light at normal incidence. Therefore, only the case of transverse magnetic (TM) polarized light is discussed in this paper.

The related structural parameters are as follows: structure period (*P*), incident angle (*θ*), nanowell width of top layer metallic groove (*w*), thickness of nanowell (*h*) and PMMA thickness (*g*). The refractive index of PMMA, prism and ambient medium are represented by *n*_a_ (1.46), *n*_b_ (1.513) and *n* (1.33), respectively. A TM-polarized plane wave (magnetic field along metal nanowells) irradiated through the structure from prism substrate. We analyzed optical characteristics of this sensor using COMSOL Multiphysics (5.3 Version, Comsol company, Stockholm, Sweden), commercial software based on finite element methods (FEM), to obtain the reflection spectra and electromagnetic field distributions. In this simulation, period boundary conditions were used along the *x* and *y* direction, and perfectly matched layers were applied in the *z* direction. The accuracy of the numerical results was mainly tested by adjusting the size of triangular grids and relative tolerance of the solver. The size of non-uniform triangular grids was used with a maximum mesh size of 10 nm and minimum mesh size of 1 nm. The relative tolerance of the solver was set in the range from 0.001 to 0.000001. Moreover, the results were further confirmed using the finite difference time domain (FDTD) algorithm (Lumerical FDTD Solutions, Inc., Vancouver, Canada). A Drude–Lorentz model defined the frequency dispersion constant of gold as [40]:

(2) $$\varepsilon_{m}(\omega )=\varepsilon_{r}-\frac{\omega_{p}^{2}}{\omega (\omega +i\gamma )}-\frac{\Delta \varepsilon \Omega^{2}}{\omega^{2}-\Omega^{2}+i\omega \Gamma }$$

where $\omega_{p}$ is the plasma frequency, collision frequency associated with energy loss *γ* is 9.874 × 10^14^ rad/s, oscillator strength Ω is 4.077 × 10^15^ rad/s, spectral width of Lorentz oscillator is 6.578 × 10^15^ rad/s, weighting of factor Δε is 1.090 and $\varepsilon_{r}$ is 5.967. Moreover, the designed structure can be fabricated by a combination of electron beam evaporation and electron beam lithography. The optical properties and sensing response of the fabricated structure can be measured with an angle-resolved macrospectroscopy system.

## 3. Results and Discussion

**Figure 2:**
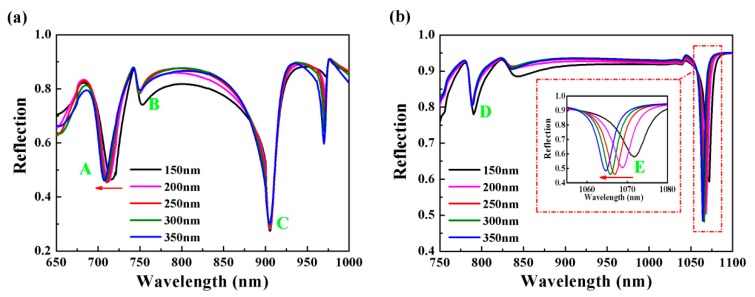
Reflection spectra for different thickness of polymethyl methacrylate (PMMA) *g* with incident angle (**a**) *θ* = 65° and (**b**) *θ* = 30°.

In order to investigate the sensing performance of the proposed double-layer plasmonic groove and the physical mechanism of its resonant modes under different incident angles, we firstly investigated the influence of structural parameter changes on resonance modes. Figure 2 shows the reflection spectra of the double-layer plasmonic groove at *θ* = 65° and *θ* = 30° for different thickness (*g*) of the PMMA, with structure parameters *P* = 1000 nm, *h* = 100 nm and *w* = 50 nm. Three resonant dips A (*λ*_1_ = 710.5 nm), B (*λ*_2_ = 748.5 nm) and C (*λ*_3_ = 905 nm) were observed at 65°, and there were two resonant dips D (*λ*_4_ = 788.5 nm) and E (*λ*_5_ = 1066.8 nm) at 30°, although there were four resonances at the incident angle of 60° and three resonances for incident angle 30°. However, the sensing performance of the resonance at the wavelength of ~971.5 nm with incident angle 65° was not good. In addition, the depth of resonant dip at ~830 nm with incident angle 30° was very small, resulting in lower sensing performance. Therefore, the sensing performance of these two resonant modes has not been considered in the manuscript. According to Figure 2a, the effect of the thickness of PMMA on each resonance dip was different. As the thickness of the dielectric layer increased, dip A underwent a blue shift, but the positions of dips B and C hardly transformed. When the incident angle changed to 30°, it can be seen from Figure 2b that the position and intensity of dip D were nearly unchanged. However, there was a blue shift at the wavelength position of dip E. After a comprehensive comparison, the sharpness of the resonance value was better with PMMA thickness *g* = 250 nm. Unless stated, the value of PMMA thickness will remain constant in the following text.

**Figure 3:**
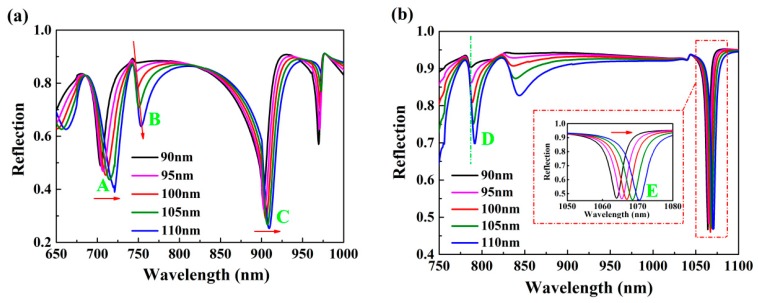
The effect of the thickness of gold layer *h* on reflection spectra under incident angle (**a**) 65° and (**b**) 30°.

Figure 3 shows the influence of the thickness *h* on reflection spectra with other structural parameters unchanged. As the thickness *h* increased, all dips had red shifts and the depths of them increased. However, compared with other dips, the wavelength shift of dip C was relatively small in Figure 3a. Figure 3b illustrates that the thickness *h* had an important effect on the intensity of dip D. Therefore, the thickness *h* was one of the crucial factors affecting the line width of dips C and E. When the thickness *h* was small, the evanescent waves passed through the gold layer and entered the adjacent dielectric layer, which caused the energy of incident light to not be coupled into the surface maximally, affecting the excitation of surface plasmon. On the contrary, when the thickness *h* increased, the attenuation of the evanescent wave in the gold film layer increased, which generated the coupling between incident light, and the surface plasmon wave became weak. For dips B and D, the choice of different *h* had an obvious effect on the resonance intensity. For the case of dips A and C and E, the reflection intensity became large and the FWHM increased with the increase of the thickness *h*, which is disadvantageous for wavelength modulation sensing. Therefore, the FWHM and reflection intensity are two important factors for the selection of structural parameters. In this paper, the thickness of gold layer was selected to be 100 nm in consideration of the sensing performance.

**Figure 4:**
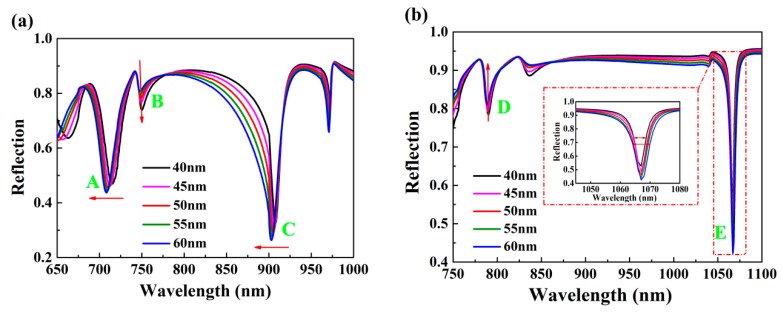
The effect of different nanowell width *w* on resonant dips under incident angle (**a**) 65° and (**b**) 30°. Inset in (**b**) clearly shows the change of line width of dip E for different nanowell width *w*.

Figure 4 depicts the influence of nanowell width *w* on reflectance spectra of the double-layer plasmonic grooves. As shown in Figure 4a, both dip A and dip C had a blue shift with increasing nanowell width *w* at *θ* = 65°. The filling factor *f* is defined as the ratio of nanowell width *w* and structure period *P* (i.e., *f* = *w/P*). When nanowell width *w* became large, that is, the filling factor *f* increased, the electric field intensity around the upper layer groove was weakened, leading to the appearance of the blueshift of above two dip. It was also found that the reflection intensity of dip B had an increase, but the reflection intensities of both dip A and dip C were gradually reduced. The above phenomenon reflects that the coupling mechanisms for three resonant dips are different. When the incident angle was reduced to 30°, the reflection intensity of dip D became large as nanowell width *w* increased, which is opposite the case of dip E (Figure 4b). Compared to the case of incident angle 65°, the amount of wavelength shift was relatively small. The appearance of both dip B and dip D was the result of the vanishing wave excited by the underlying gold, and the width of *w* directly affected the strength of the coupling and the magnitude of reflection (Figures 6a and 7c). Furthermore, as nanowell width *w* increased, the linewidth of dip A remained almost unchanged, but the linewidths of both dip C and dip E became wide.

**Figure 5:**
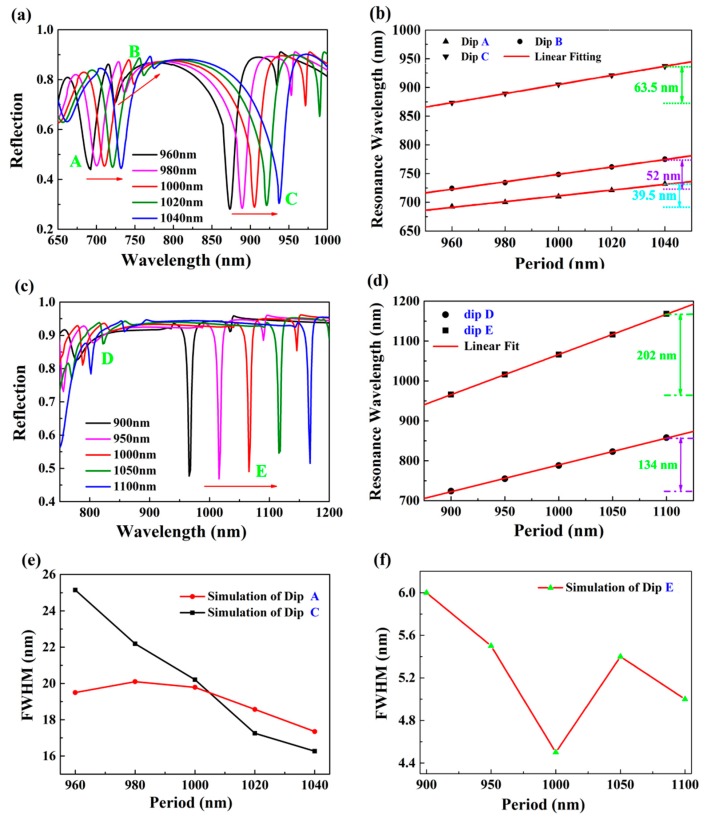
The influence of structural period *P* on resonant dips. For the case of incident angle 65°, (**a**) reflection spectra, (**b**) the relationship between wavelength position of resonant dips and period *P*, and (**e**) the effect of period *P* on line width of dips A and C. For the case of incident angle 30°, (**c**) reflection spectra, (**d**) the relationship between wavelength position of resonant dips and period *P*, and (**f**) the effect of period *P* on line width of dip E.

Figure 5 depicts the effect of different structural periods *P* on the reflection spectrum at *θ* = 65° and 30°. Obviously, all dips had red shift as period *P* increased (Figure 5a,c). This trend was exactly opposite of nanowire width *w*. According to surface plasmon excitation condition of plasmonic groove, the increase of the period caused a red shift of the wavelength of resonance dip. This shows that the appearance of resonant dip is related to the excitation of SPP mode. As can be seen clearly from Figure 5b,d, the resonant dip and the period satisfy a linear relationship. In addition, we also investigated the variation of FWHM with the period in Figure 5e,f. When structure period *P* had a change in the range from 960 nm to 1040 nm, the FWHM of dip A varied from 18 nm to 20 nm, and there was a change from 25 nm to 17 nm for the linewidth of dip C. Moreover, compared to the case of incident angle 65°, the FWHM of resonant dip at 30° was not sensitive to the change of structural period, which is favorable in the fabrication of proposed structure.

**Figure 6:**
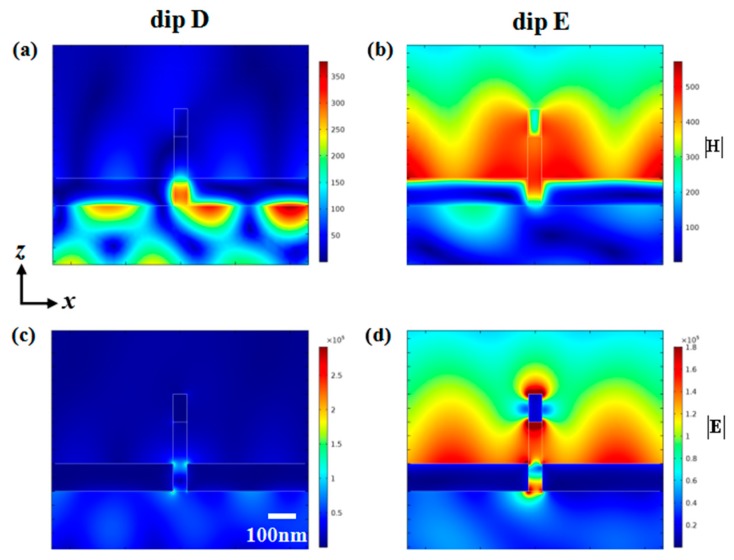
Near-field distribution at resonant dips when the incident angle is 30°. Magnetic field distribution of (**a**) dip D and (**b**) dip E. Electric field distribution of (**c**) dip D and (**d**) dip E.

We further made an investigation of above optical phenomenon of the proposed structure. Figure 6a,c illustrate the magnetic field and electric field distributions at dip D, respectively. It can be seen that the magnetic field was mainly concentrated on the interface between the gold groove and the prism, and a portion of the energy entered the dielectric layer. The field intensity was exponentially attenuated in the vertical direction and periodically distributed in the horizontal direction. These phenomena are consistent with near-field distribution of surface plasmon mode for the groove structure [23,41]. Observing the magnetic field distribution of dip E, it was completely different from dip D in Figure 6b. At dip E, the energy was localized at the interface between groove and air and the upper portion of the dielectric layer. The electric field distribution of Figure 6d shows that electric dipole mode of upper gold nanowell is excited. Therefore, resonant dips came from mutual coupling of different modes. Furthermore, the near-field distribution of dip E was mainly localized on the upper surface of the groove. So dip E was more sensitive to changes in refractive index of ambient environment than that of dip D.

**Figure 7:**
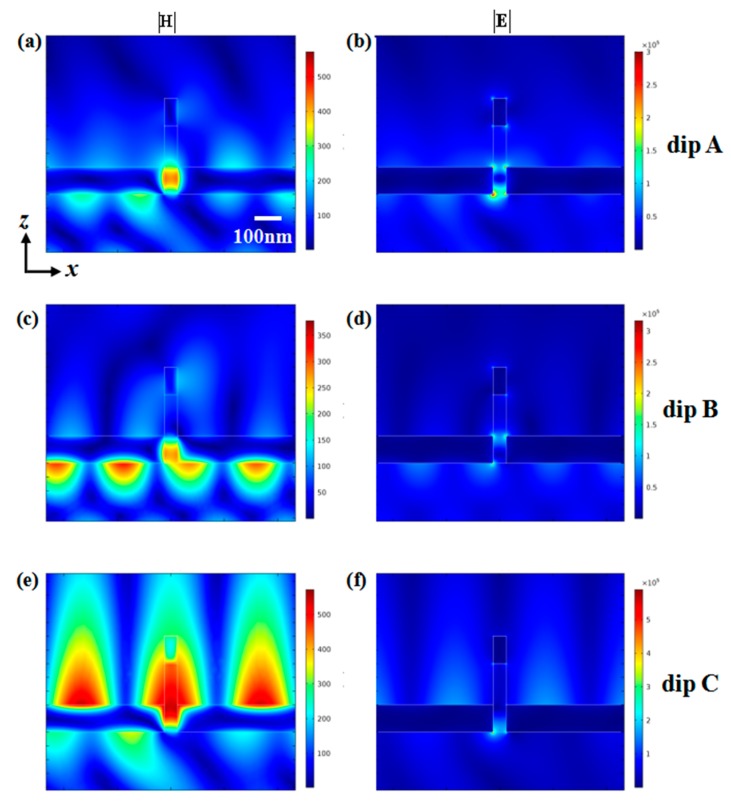
Near-field distribution at resonant dips when the incident angle is 65°. (**a**) Magnetic field distribution and (**b**) electric field distribution at resonance dip A. (**c**) Magnetic field distribution and (**d**) electric field distribution at resonance dip B. (**e**) Magnetic field distribution and (**f**) electric field distribution at resonance dip C.

To further understand the physical properties of resonance dips of A, B and C at incident angle 65°, their electromagnetic distributions are shown in Figure 7. As shown in Figure 7a,b, the electromagnetic field of dip A was mainly concentrated in the dielectric layer while a small portion was distributed in the upper and lower surface of the bottom-layer groove. The near-field distribution at the surface of the bottom-layer groove clearly conforms to the characteristics of SPP mode. The near-field distribution in the dielectric layer was due to zero-order cavity mode to be excited [37], which caused a portion of the electromagnetic field to be coupled to gold nanowell. Compared with that of dip A, it can be seen from Figure 7c,d that the field distribution at dip B was significantly weaker and mainly localized on the PMMA slit and the lower surface of bottom-layer groove. As shown in Figure 7e,f, the near-field distribution at dip C depicts that the electromagnetic field was mainly concentrated on the upper surface of the bottom-layer groove and the dielectric layer, that is, the SPP mode and the cavity mode were coupled with each other. The introduction of top-layer nanowell caused the electromagnetic field to be localized in the dielectric layer, while the field intensity of upper nanowell was very weak, which is the reason why the depth of the reflection dip was the largest. In addition, the electromagnetic field was nearly distributed on the upper surface of the double-layer groove, so refractive index sensitivity at dip C was largest.

**Figure 8:**
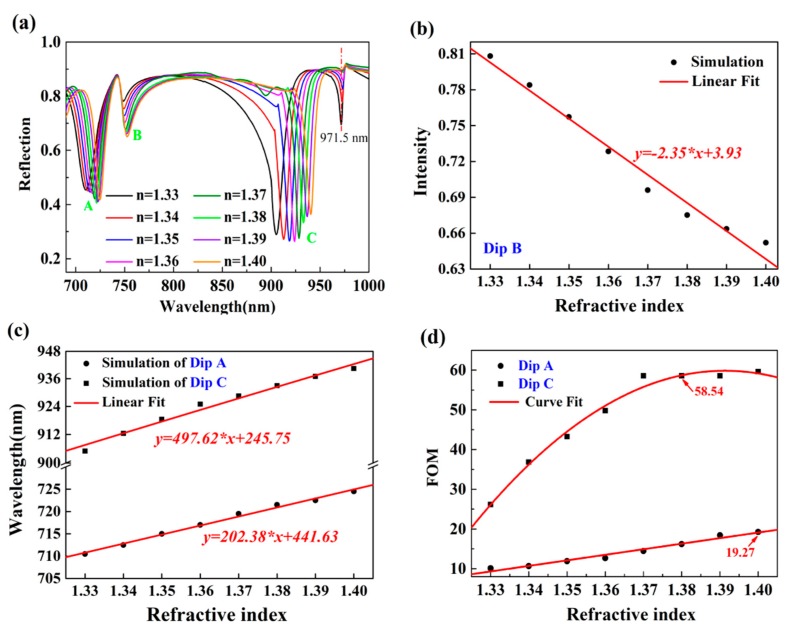
Sensing performances of proposed double-layer plasmonic grooves at incident angle 65°. (**a**) The relationship of sample refractive index (RI) and relative intensity of dip B. (**b**) The relationship of sample RI and resonance wavelength of both dips A and C. (**c**) Figure of merits (FOM) for resonant modes of both dips A and C.

To quantitatively evaluate the sensing performance of the proposed structure, we analyzed two major criteria in sensing detection, refractive index (RI) sensitivity and FOM. Here, FOM is defined as the ratio of bulk refractive index sensitivity and full width at half maximum, i.e., FOM = *S*/FWHM. Figure 8a shows the evolution of reflection spectra for the analyte on the surface of structure surface with a refractive index range of 1.33–1.40 for the incident angle of 65°. Figure 8b,c illustrate the RI sensitivity of these three resonant dips, which is defined as the change of resonant wavelength or resonant intensity with respect to the bulk RI changes. In this structure, the RI sensitivities were S_A_ = 202.38 nm/RIU, S_B_ = 2.35 RIU^−1^ and S_C_ = 497.62 nm/RIU, respectively. They also had good linear approximations in the whole RI range. Compared with dip C, the sensitivity of dip A was significantly smaller. As shown in Figure 7, the electromagnetic field of dip C was mainly localized in the upper of gold layer, and could sufficiently contact the analyte, so the sensitivity was high. Dip A was excited by zero-order cavity mode of the bottom-layer groove, which resulted in lower sensitivity. Figure 8d depicts the FOM of both dip A and dip C. The FWHM of dip A could be as small as 10.5 nm, while dip C was 8.5 nm. At these two resonance dips, as refractive index of analyte increased, the FWHM gradually decreased, that is, the FOM became larger. The maximum of FOM at dip A was 19.27, and dip C was 58.54. It was observed that at resonant dip C, the FWHM and FOM remained constant in the range of 1.37–1.40. The design of the structure not only reduced the detection limit but also realized multiple-channel measurement.

**Figure 9:**
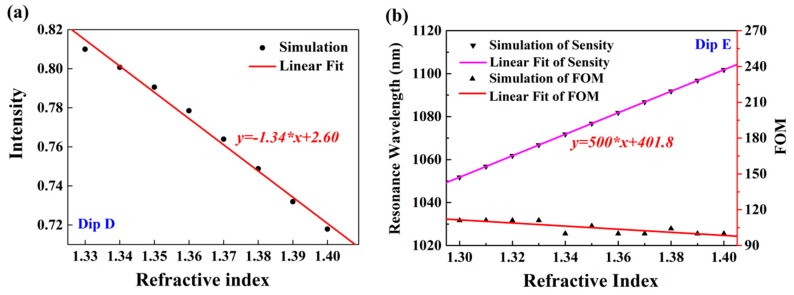
Sensing performances of proposed double-layer plasmonic grooves at incident angle 30°. (**a**) The relationship of the intensity of dip D and sample RIs. (**b**) The relationship of wavelength positions of dip E and sample RIs, and corresponding FOM.

In addition, we also investigated the sensing performance of proposed double-layer plasmonic grooves at incident angle 30°. Furthermore, reflection intensity at dip D changed significantly with the increase of ambient RIs, and the refractive index sensitivity S_D_ was about 1.34 RIU^−1^, as shown in Figure 9a. Comparing the electric field distributions at dip B and dip D, it was found that both of them were caused by SPPs. Owing to the orders of SPPs being different when plasmonic grooves are irradiated at different incident angles, which is evident in the electric field diagram, the intensity index sensitivity of dip B was greater than that of dip D. Compared with the case of incident angle 65°, this structure obtained a high RI sensitivity of S_E_ = 500 nm/RIU at incident angle 30°. Moreover, the FWHM of dip E was extremely narrow and could be stably maintained at 4.5 nm in the RI range from 1.3 to 1.4. Therefore, FOM was up to 111.11 and had a good linear approximate, as shown in Figure 9b. The above two values indicate that the proposed structure can achieve important applications in sensing detection. Compared with both dips A and C, the FOM of dip E was increased by 5.8 times and 1.9 times, respectively. Thus, two-channel measurements can be achieved at the incident angle 30°, and the structure can achieve three resonance measurements at the incident angle 65°.

## 4. Conclusions

In summary, we have theoretically demonstrated a high sensitivity and multi-resonance sensor based on a double-layer metallic grooves configuration. When the incident angle is 65°, the reflection spectrum has three resonance dips, and the corresponding sensitivities are 2.35 RIU^−1^, 202.38 nm/RIU and 497.62 nm/RIU in the RI range from 1.33 to 1.40, and FOM is up to 19.27 and 58.54, respectively. The excitation of resonant dips mainly depends on the coupling between SPP mode and cavity mode. Changing the angle to 30°, the metal groove achieves dual resonance measurement. The refractive index of the resonance dip is 500 nm/RIU at 1050 nm, FWHM maintains at 4.5 nm, and FOM attains 111.11. The occurrence of this narrow band is due to the coupling between SPP mode and dipole resonance. Moreover, by employing the coupling between different modes under oblique incidence, our proposed structure achieves the purpose of free switching between two or more resonant sensors. In addition to the simple preparation process, the groove has a high quality factor, effectively reducing the detection limit in biosensing. This work is valuable for the developing of multi-resonance and high-performance sensors. Notably, the optimization of the structure parameter in this paper aims to obtain resonant dip with narrow line width and large depth, which are robust in experiment fabrication. In addition to a robust preparation process, the groove has a high quality factor, effectively reducing the detection limit in biosensing. This work is valuable for the developing of multi-resonance and high-performance sensors.
